# Multiparametric Modelling of Survival in Pancreatic Ductal Adenocarcinoma Using Clinical, Histomorphological, Genetic and Image-Derived Parameters

**DOI:** 10.3390/jcm9051250

**Published:** 2020-04-25

**Authors:** Georgios A. Kaissis, Friederike Jungmann, Sebastian Ziegelmayer, Fabian K. Lohöfer, Felix N. Harder, Anna Melissa Schlitter, Alexander Muckenhuber, Katja Steiger, Rebekka Schirren, Helmut Friess, Roland Schmid, Wilko Weichert, Marcus R. Makowski, Rickmer F. Braren

**Affiliations:** 1 Department of Diagnostic and Interventional Radiology, School of Medicine, Technical University of Munich, 81675 Munich, Germany; 2 Department of Computing, Faculty of Engineering, Imperial College of Science, Technology and Medicine, London SW7 2BU, UK; 3 Institute for Pathology, School of Medicine, Technical University of Munich, 81675 Munich, Germany; 4 German Cancer Consortium, Partner Site Technical University of Munich, D-69120 Heidelberg, Germany; 5 School of Medicine, Surgical Clinic and Policlinic, Technical University of Munich, 81675 Munich, Germany; 6 Department of Internal Medicine II, School of Medicine, Technical University of Munich, 81675 Munich, Germany

**Keywords:** pancreatic ductal adenocarcinoma, survival analysis, multiparametric modelling, genetics, molecular phenotyping, image-derived features

## Abstract

Rationale: Pancreatic ductal adenocarcinoma (PDAC) remains a tumor entity of exceptionally poor prognosis, and several biomarkers are under current investigation for the prediction of patient prognosis. Many studies focus on promoting newly developed imaging biomarkers without a rigorous comparison to other established parameters. To assess the true value and leverage the potential of all efforts in this field, a multi-parametric evaluation of the available biomarkers for PDAC survival prediction is warranted. Here we present a multiparametric analysis to assess the predictive value of established parameters and the added contribution of newly developed imaging features such as biomarkers for overall PDAC patient survival. Methods: 103 patients with resectable PDAC were retrospectively enrolled. Clinical and histopathological data (age, sex, chemotherapy regimens, tumor size, lymph node status, grading and resection status), morpho-molecular and genetic data (tumor morphology, molecular subtype, tp53, kras, smad4 and p16 genetics), image-derived features and the combination of all parameters were tested for their prognostic strength based on the concordance index (CI) of multivariate Cox proportional hazards survival modelling after unsupervised machine learning preprocessing. Results: The average CIs of the out-of-sample data were: 0.63 for the clinical and histopathological features, 0.53 for the morpho-molecular and genetic features, 0.65 for the imaging features and 0.65 for the combined model including all parameters. Conclusions: Imaging-derived features represent an independent survival predictor in PDAC and enable the multiparametric, machine learning-assisted modelling of postoperative overall survival with a high performance compared to clinical and morpho-molecular/genetic parameters. We propose that future studies systematically include imaging-derived features to benchmark their additive value when evaluating biomarker-based model performance.

## 1. Introduction

Pancreatic ductal adenocarcinoma (PDAC), despite its relative rarity, remains among the deadliest tumor entities in the developed world. For instance, PDAC is the 4th leading cause of cancer-related death while representing only 3% of newly diagnosed cancer cases in the United States [1]. A large body of research into therapeutic targets, and newly introduced therapy regimens, have hitherto not been able to improve overall PDAC patient prognosis beyond a five-year survival of about 9% [2]. Even in the setting of tumor resection and adjuvant chemotherapy [3], patients often develop therapy-resistant tumor recurrence or metachronous metastatic disease. The key driver of such therapy escape phenomena is likely to be tumor heterogeneity, both on the genetic level, with the four driver genes kras, smad4, tp53 and p16 [4,5] having been shown to lead to distinct survival outcomes [6,7]. On the molecular/histo-morphologic level, quasi-mesenchymal and epithelial tumors demonstrate a distinct chemotherapy response [8,9]. Hence, despite the overall poor prognosis in PDAC, significant differences in individual patient survival are noted. Therefore, outcome prediction and patient stratification based on available parameters represent important goals in clinical patient care. The current gold standard for tumor assessment, biopsy and histopathology, entail a major risk of misclassifying tumors due to undersampling and sampling errors. Moreover, non-invasive biomarkers have yet to reach clinical maturity. Recently however, evidence has emerged that the machine learning-based analysis of pre-therapeutic imaging can provide a decision guidance based on the non-invasive derivation of quantitative, whole-tumor characteristics [10,11]. Image pattern analysis and machine learning approaches (often termed radiomics) have yielded encouraging results in the prediction of molecular PDAC subtypes and of patient survival from magnetic resonance or computed tomography imaging [12,13]. Much of recent research, driven by the increase in attention towards the field of artificial intelligence, has concentrated exclusively on proving the superiority of radiomics to human observers or other diagnostic and prognostic parameters [14,15,16]. However, in a disease as complex as PDAC, the integration of clinical information, invasive biomarkers and imaging-derived data may provide a more comprehensive prognostic assessment of the tumor than any single modality [17]. Therefore, a fair comparison elucidating the differential contribution of each parameter type and their combined value, is warranted.

In this study, we present a multimodal, data-driven workflow including clinical information, histo-morphologic/genetic parameters and computed tomography-derived imaging biomarkers for modelling overall patient survival in the post-operative setting.

## 2. Experimental Section

The study was designed as a retrospective cohort study. The study was approved by the institutional ethics review board (Ethics Committee of the Technical University of Munich Faculty of Medicine) (Protocol Number 180/17S; date of approval: 9 May 2017), and the requirement for individual written consent was waived. All procedures were carried out in accordance with pertinent laws and regulations, as well as the Helsinki declaration. The STrengthening the Reporting of OBservational studies in Epidemiology (STROBE) checklist [18] and patient inclusion flowchart are included in the supplemental material (Table S1 and Figure S1). In brief, 177 patients with a confirmed diagnosis of PDAC were considered for inclusion. Patients without baseline computed tomography (CT) imaging, incomplete imaging, insufficient image quality (due to motion artifacts or significant beam hardening from adjacent foreign matter), patients who died sooner than six weeks after surgery or those with incomplete clinical data were excluded. A total of 103 patients were analyzed. All patients underwent tumor resection. The follow-up interval began on the 10 October 2006 and ended on the 14 April 2019. Clinical data were obtained using the hospital’s information system, and survival information was obtained from the hospital’s information system and from the national cancer registry. The genetic and histopathological analyses were carried out by the Department of Pathology of the Technical University of Munich and were available prior to the study commencement. Image-derived parameters data were collected during the analysis.

The following clinical data was obtained for all patients: sex, age at diagnosis, tumor site (head, body or tail of the pancreas), type of adjuvant chemotherapy received (gemcitabine-based vs. no chemotherapy), time to progression, type of progressive disease (local recurrence, metastasis, both), first-line chemotherapy after progression (gemcitabine, FOLFIRINOX, none/best supportive care) and overall survival time including censoring.

The following histopathological data was obtained from formalin-fixed paraffin embedded tumor specimens: tumor size (pT1/2 vs. pT3/4), lymph node status (pN0 vs. pN+), grading (G1/2 vs. G3, i.e., high vs. low grade), resection status (R0 vs. R+, including positive continuous resection margins) and molecular subtypes according to Muckenhuber et al. [19] (quasi-mesenchymal (QM) vs. non-quasi-mesenchymal (Non-QM)); and tumor morphology (conventional vs. combined) and mutational status of tp53 (wild-type vs. mutated), kras (wild-type vs. mutated), p16 (intact vs. altered) and smad4 (positive vs. loss), both as described in Schlitter et al. [20].

CT images were exported from the hospital picture archive and processed as previously described [13,21] and according to current best practices. Segmentation was performed fully automatically by in-house developed software and manually corrected by an expert observer. Image-derived features were extracted using PyRadiomics version 2.2.0 [22] with standard settings. 1409 image features were extracted. Features with a variance of less than 1e-5 were excluded, yielding 1384 radiomic features which were included in the analysis. Feature values were normalized to the (0, 1) interval. A detailed description of the radiomic extraction process can be found in the supplement (File S1). The PyRadiomics extraction settings file (.yml) can also be found in the supplement (File S2).

To assess the differential contribution of each parameter category, the following four parameter groups were analyzed: (1) clinical and standard histopathological data (age, sex, chemotherapy, pT, pN, G, R), (2) morpho-molecular and genetic data (tumor morphology, molecular subtype, and tp53, kras, smad4 and p16 genetics), (3) image-derived features and (4) all features combined.

Due to the very large number of features compared to the cohort size, an unsupervised machine learning-based dimensionality reduction and feature selection were performed as follows: linear principle component analysis (PCA) was employed for radiomic features with the aim of capturing 99% of the parameter variance. A univariate pre-selection was performed on all features using the log-rank-test statistic with a cut-off of *p* < 0.1.

The Cox Proportional Hazards model was fit to each parameter group separately at first to assess the model concordance index (CI) after asserting that the proportional hazards assumptions were met. Parameters which did not meet the proportional hazards assumptions were excluded from the final analysis of the combined features. For testing the predictive performance, a five-fold hold-out cross-validation was employed in all analyses, and the concordance indexes across the five folds were averaged. 95% confidence intervals were calculated using 100-fold bootstrap resampling. All statistical analyses were performed in Python v. 3.7.6. using the packages lifelines and scikit-learn. A two-sided significance level of *p* < 0.05 was chosen.

## 3. Results

A total of 103 patients were included in the study. Their clinical characteristics are summarized in Table 1.

Of the 103 patients included, 51 patients experienced a progressive disease during the observation period, and of these 14 (27%) experienced local recurrence, 29 (57%) liver metastasis and 8 (16%) both. Of those patients, 22 (43%) received first-line gemcitabine, 13 (25%) received first-line FOLFIRINOX and 16 (31%) received best supportive care. The median progression-free survival was 7.3 months. The disease progression (experienced vs. did not experience) or type of treatment after progression were not significantly associated with overall survival and are thus not included in the final analysis.

The Cox proportional hazards method was used for modelling the overall survival using each of the parameter categories. The median overall survival was 16.6 months, and 19/103 patients (18.4%) were censored in the final survival analysis.

**Figure 1:**
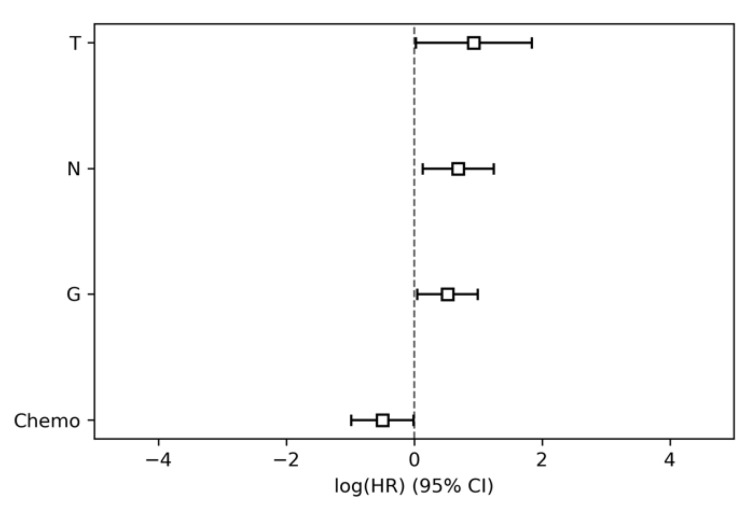
Hazard ratios and 95% confidence intervals of parameter group 1 (clinical parameters). HR, hazard ratio; T, tumor size; N, nodal status; G, tumor grading; CI, confidence interval.

The modelling of parameter group 1, i.e., the clinical and standard histopathological data (age, sex, chemotherapy, pT, pN, G, R) achieved a CI of 0.63 (95% conf. int. 0.60–0.66). The nodal status (pN), tumor grading (G), tumor size (pT) and chemotherapy regimen were significantly associated with overall survival (Table 2, Figure 1).

**Figure 2:**
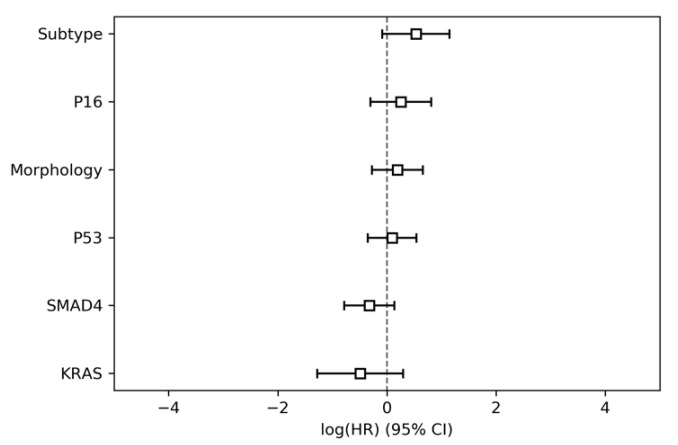
Hazard ratios for parameter group 2 (histo-morphologic and genetic parameters).

In parameter group 2, i.e., the morpho-molecular and genetic data (tumor morphology, molecular subtype, and pt53, kras, smad4 and p16 genetics), the tumor morphology did not meet the proportional hazard assumptions and was therefore excluded from the combined analysis. None of the parameters were significantly associated with overall survival (Table 3, Figure 2). The model resulted in a CI of 0.53 (95% conf. int. 0.47–0.59).

**Figure 3:**
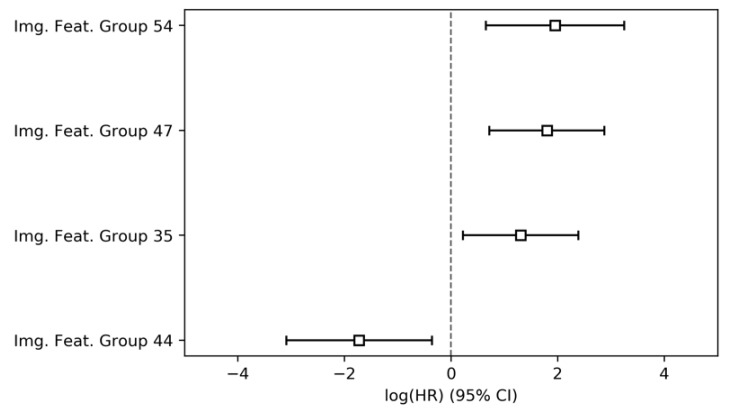
Hazard ratios and 95% confidence intervals of parameter group 3 (image-derived parameters).

The principal component analysis of the imaging features, i.e., parameter group 3, resulted in 71 unique image-derived feature groups representing 99% of the total parameter variance. Their modelling was preceded by a univariate pre-selection, after which eight image feature groups were retained for inclusion in the multivariate model. Of these, four were significantly associated with overall survival (Table 4, Figure 3), three negatively and one positively. The multivariate model achieved a CI of 0.65 (95% conf. int. 0.62–0.69).

**Figure 4:**
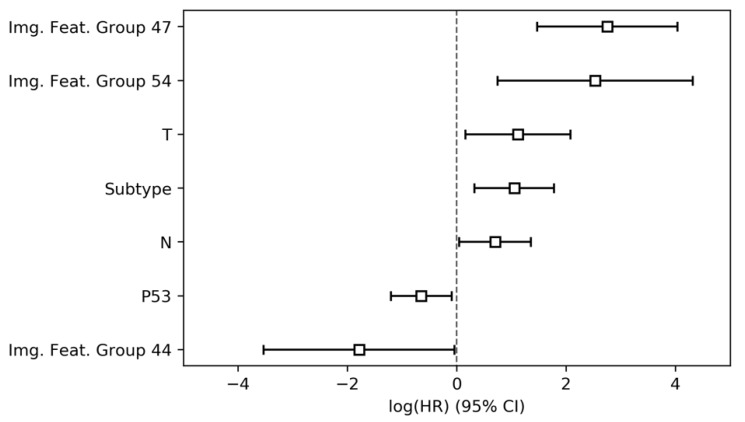
Hazard ratios and 95% confidence intervals of parameter group 4 (all parameter groups combined).

Finally, to assess the predictive performance of the combined feature set and model feature interactions, the features from parameter groups 1 through 3 which were found to obey proportional hazards assumptions were jointly included in a Cox proportional hazards model. In total, seven features, namely three image-derived parameter groups, the tumor subtype, tp53 mutation status, tumor size (pT) and nodal status (pN), were found to significantly and independently predict overall survival (Table 5, Figure 4). The final combined model achieved a CI of 0.65 (95% conf. int. 0.60–0.69).

## 4. Discussion

We here present the results of a comprehensive multiparametric analysis for the prediction of overall patient survival in a group of resected PDAC patients. Our results underscore the potential of image-derived biomarkers compared to both conventional histopathological and novel molecular, morphologic and genetic phenotyping in the context of patient risk stratification in PDAC.

In our previous work, we reported on survival prediction based on imaging biomarkers alone and developed models for the stratification of molecular phenotypes, therapy response and patient survival from computed tomography and diffusion-weighted magnetic resonance images [12,13,21]. Our current work expands on these efforts by including broadly available clinical parameters and unestablished, novel biomarkers in multivariate survival models. Interestingly, both conventional clinical parameters, such as tumor stage, nodal status and adjuvant chemotherapy, and the imaging-derived biomarkers introduced in this study outperform morpho-molecular and genetic parameters alone. We believe this to be an effect of the parameter distribution in this cohort and the overall cohort size. Both previous work and our own multivariate analysis have shown genetic parameters [20] and molecular tumor subtypes [8,19] to significantly predict survival. Furthermore, we believe sampling errors of the histo-morphological and genetic workup to reduce the representativeness of invasive biomarkers, and we thus highlight the necessity for a whole tumor analysis. Despite the fact that imaging-derived biomarkers do not outperform other parameters (0.63 versus 0.65 CI), they are justified due to their non-invasive nature, which applies especially to the palliative and neo-adjuvant settings.

The recent study by Zhang et al. [17] showed a similar survival model concordance index of around 0.65 on validation data by applying convolutional neural networks (CNN) to CT imaging data. The non-inferiority of our approach compared to the more advanced CNN algorithm might be a result of the limited sample size, a common problem in current imaging studies [23].

To our knowledge, this is the first study to systematically evaluate a broad range of currently available PDAC risk biomarkers. The sample size studied, and the absence of an external validation cohort, are a limitation of our study but also an immediate consequence of the high financial and personnel cost of the extensive morpho-molecular and genetic workup. Considering the lack of re-imbursement for the detailed tissue analyses presented, biomarkers derived from routine clinical data—including imaging—are a viable option in the setting of limited healthcare resources. Nevertheless, our results should be tested prospectively to ascertain their external validity.

## 5. Conclusions

In summary, this study presents a further step towards the validation of multi-parametric analyses in the context of clinical patient care. Two conclusions arise from our results: A side-by-side direct comparison of whole tumor tissue and imaging-data derived features will be required to adequately compare the true predictive performance of imaging against morpho-molecular tumor constituents. Furthermore, for multiparametric data integration—as demonstrated in our work—to reach clinical maturity, multi-centric cooperation will be required to attain sufficient sample sizes.

## Figures and Tables

**Table 1:** Clinical and histopathological characteristics of the 103 patients included in the study.

	*n* = 103	%
Sex		
Male	59	57.2
Female	44	42.8
Age		
Mean in years	67.3	
Range	32–88	
Subtype		
QM	16	15.5
Non-QM	87	84.5
pT		
1	1	0.9
2	10	9.7
3	80	77.7
4	12	11.7
pN		
0	30	30.1
1	73	70.9
Grading		
1	5	4.9
2	44	42.8
3	54	52.3
Resection status		
0	53	51.4
1	50	48.6
Morphology		
Conventional	55	53.4
Combined	48	46.6
Adjuvant Chemotherapy		
Gemcitabine	55	53.3
Did not receive	48	46.7
Tumor Location		
Head	71	68.9
Body	19	18.4
Tail	13	12.7
TP53		
Wild type	21	20.3
mutated	82	79.7
KRAS		
wildtype	9	8,8
mutated	94	91.2
CDKN2A/p16		
intact	19	81.5
altered	84	18.5
SMAD4		
intact	41	39.2
altered	62	60.8

QM, quasi-mesenchymal; pN, nodal status; pT, tumor size.

**Table 2:** Results of the Cox proportional hazards modelling for parameter group 1 (clinical parameters).

	HR	Lower 95% Conf. Int.	Upper 95% Conf. Int.	*p*
T	2.54	1.03	6.27	0.04
N	1.99	1.14	3.47	0.02
G	1.68	1.05	2.71	0.03
R	1.37	0.87	2.16	0.18
Sex	1.04	0.64	1.71	0.86
Age	1.0	0.98	1.02	0.85
Location	0.93	0.56	1.54	0.77
Adjuvant Chemo	0.61	0.37	0.99	0.04

HR, hazard ratio; T, tumor size; N, nodal status; G, tumor grading; R, resection status.

**Table 3:** Results of the Cox proportional hazards modelling for parameter group 2 (histo-morphologic and genetic parameters).

	HR	Lower 95% Conf. Int.	Upper 95% Conf. Int.	*p*
Subtype	1.69	0.92	3.13	0.09
P16	1.28	0.74	2.24	0.38
Morphology	1.21	0.76	1.92	0.42
P53	1.09	0.7	1.71	0.7
SMAD4	0.72	0.46	1.14	0.16
KRAS	0.61	0.28	1.34	0.22

**Table 4:** Results of the Cox proportional hazards modelling for parameter group 3 (image-derived parameters).

	HR	Lower 95% Conf. Int.	Upper 95% Conf Int.	*p*
Img. Feat. Group 54	7.0	1.91	25.61	<0.001
Img. Feat. Group 47	6.03	2.05	17.72	<0.001
Img. Feat. Group 35	3.67	1.24	10.87	0.02
Img. Feat. Group 56	3.46	1.01	11.81	0.05
Img. Feat. Group 67	1.33	0.44	4.02	0.62
Img. Feat. Group 21	0.58	0.15	2.17	0.42
Img. Feat. Group 27	0.34	0.08	1.39	0.13
Img. Feat. Group 44	0.18	0.05	0.7	0.01

**Table 5:** Results of the Cox proportional hazards modelling for parameter group 4 (all parameters combined).

	HR	Lower 95% Conf Int.	Upper 95% Conf Int.	*p*
Img. Feat. Group 47	15.68	4.35	56.45	<0.001
Img. Feat. Group 54	12.56	2.11	74.81	<0.001
Img. Feat. Group 35	3.08	0.86	11.04	0.08
T	3.05	1.17	7.96	0.02
Subtype	2.86	1.38	5.92	<0.001
N	2.01	1.04	3.87	0.04
Img. Feat. Group 56	1.66	0.37	7.42	0.51
P16	1.49	0.82	2.73	0.19
G	1.33	0.79	2.23	0.28
R	1.28	0.71	2.3	0.41
Location	1.08	0.6	1.97	0.79
Sex	1.01	0.59	1.72	0.97
Age	1.0	0.97	1.02	0.82
Img. Feat. Group 67	0.85	0.23	3.1	0.81
SMAD4	0.83	0.48	1.41	0.48
Chemo	0.67	0.37	1.23	0.2
KRAS	0.67	0.24	1.88	0.45
Img. Feat. Group 21	0.58	0.13	2.47	0.46
P53	0.52	0.3	0.91	0.02
Img. Feat. Group 27	0.35	0.07	1.69	0.19
Img. Feat. Group 44	0.17	0.03	0.96	0.04

## Supplementary Materials

The following are available online at https://www.mdpi.com/2077-0383/9/5/1250/s1: Table S1: STROBE statement checklist; Figure S1: patient inclusion flowchart; File S1: Image-derived feature extraction details, File S2: PyRadiomics parameter file.
